# Acute hepatitis in a woman following excessive ingestion of an energy drink: a case report

**DOI:** 10.1186/1752-1947-5-227

**Published:** 2011-06-22

**Authors:** Abhirami Vivekanandarajah, Shirley Ni, Alain Waked

**Affiliations:** 1 Department of Medicine, Staten Island University Hospital, 475 Seaview Avenue, Staten Island, NY 10305, USA

## Abstract

**Introduction:**

The consumption of energy drinks has increased significantly. We report the case of a patient who presented to our hospital with jaundice, abdominal pain, and markedly increased liver transaminases likely due to the increased consumption of an energy drink. To the best of our knowledge, this is the first case report in the literature linking the development of acute hepatitis to the consumption of an energy drink.

**Case presentation:**

A 22-year-old Caucasian woman presented to our hospital with epigastric pain, nausea, vomiting, and low-grade fever. She had been drinking 10 cans of an energy drink daily for two weeks prior to presentation. Her physical examination revealed mild epigastric tenderness. Her initial blood tests revealed elevated alanine aminotransferase, aspartate aminotransferase, and total bilirubin. A computed tomographic scan of the abdomen and pelvis was normal, and the patient was discharged to home. She returned to the Emergency Department of our hospital with worsening pain and new-onset jaundice. This time her physical examination revealed epigastric tenderness and icteric sclera. Her aspartate aminotransferase, alanine aminotransferase, and international normalized ratio were markedly elevated. Further radiological studies were non-specific, and she was admitted to our hospital with a diagnosis of acute hepatitis. Her viral serology and toxicology screens were negative. The patient was treated supportively and was discharged after resolution of her symptoms and a marked decrease in her liver enzymes.

**Conclusion:**

The development of acute hepatitis in this patient was most likely due to the excessive ingestion of an energy drink, and we speculate that niacin was the culprit ingredient.

## Introduction

A large number of people are consuming numerous herbal supplements and energy drinks. To date, no cases have linked energy drinks to hepatitis. In this case report, we presume that the development of acute hepatitis was due to the increased consumption of an energy drink.

## Case presentation

A 22-year-old healthy Caucasian woman presented to the Emergency Department (ED) of our hospital with epigastric pain, nausea, vomiting, and low-grade fever. During the two weeks before her presentation to the Emergency Department, she had been consuming about 10 cans of an energy drink daily. She denied ingestion of alcohol, other medications, or illicit drugs. Her diet was unchanged. Initially, her physical examination was unremarkable except for mild epigastric tenderness. Blood tests revealed an aspartate aminotransferase (AST) level of 171U/l, an alanine aminotransferase (ALT) level of 216U/l, and a total bilirubin level of 1.7 mg/dl. A computed tomographic scan of her abdomen and pelvis with oral and intravenous contrast enhancement revealed no abnormalities, and she was discharged to home from the ED. The following day she returned with worsening pain and new-onset jaundice. Her examination was unchanged, with the exception of icteric sclera. Her AST, ALT, and international normalized ratio values were 7709U/l, 7533U/l, and 1.6, respectively. Her total bilirubin, direct bilirubin, and albumin levels were 3.5 mg/dl, 1.9 mg/dl and 3.8 g/dl, respectively. Her γ-glutamyl transpeptidase level was 29U/l, her acetaminophen level was undetectable, and her ammonia level was 23 μM/l. Her alkaline phosphatase, amylase, and lipase levels were normal. Her toxicology screen was negative. Ultrasound of the abdomen showed non-specific borderline gallbladder wall thickening. She was admitted with a diagnosis of acute hepatitis. Serology tests for Epstein-Barr virus; cytomegalovirus; and hepatitis A, B, C, and E virus were negative. An autoimmune work-up was not performed. She received intravenous hydration and nothing by mouth initially. She continued to improve, and her diet was advanced. Four days later she was discharged to home after her symptoms had resolved. Her AST, ALT, and total bilirubin levels were 238U/l, 1947U/l, and 1.7 mg/dl, respectively, upon discharge. She returned to the medical clinic for follow-up after one month, and the ALT and AST results were 22U/l and 26U/l, respectively, at that time.

## Discussion

Consumers have been indulging in increased consumption of popular energy drinks. It is felt that these energy drinks are benign and are packed with good ingredients such as B vitamins and amino acids that do not have adverse effects.

Energy drinks contain a blend of B vitamins, the energy blend, and enzymes. The vitamins they contain include vitamin B_6_, vitamin B_3_, vitamin B_12_, and vitamin B_9_; the energy blend consists of citicoline, taurine, tyrosine, phenylalanine, malic acid, caffeine, and glucuronolactone; and the enzymes they contain include amylase, protease, cellulase, protease, and lactase (Table 1).

**Table 1:** Ingredients of the energy drink (as listed on the manufacturer's product label)^a^

Ingredients of the energy drink (serving size two fluid ounces)	Amount per serving	Daily value,^b ^%
Niacin	30 mg	150%

Vitamin B_6_	40 mg	2000%

Folic acid	400 μ g	100%

Vitamin B_12_	500 μ g	8333%

Sodium	10 mg	< 1%

Energy blend	1870 mg	Not known

^a^Other ingredients in the energy drink are purified water, natural and artificial flavors, sucralose, potassium sorbate, sodium benzoate, and ethylenediaminetetraacetic acid; ^b^daily value percentage is based on the daily intake recommended by the U.S. Food and Drug Administration.

Vitamin B_6 _is important in heme, nucleic acid, lipid, carbohydrate, and amino acid metabolism. Toxicity occurs when megadoses of vitamin B_6 _(> 500 mg/day) are ingested [1]. Symptoms of toxicity include peripheral neuropathy with deficits in a stocking-glove distribution, progressive sensory ataxia, and severe impairment of position and vibration senses. Vitamin B_6 _toxicity is not linked to hepatotoxicity. The treatment of vitamin B_6 _toxicity is to discontinue vitamin B_6 _consumption, and recovery is slow and, for some patients, incomplete.

Niacin and its derivatives are vital to cell metabolism. They have been used for decades in the treatment of patients with disturbed lipid and lipoprotein metabolism. They have been associated with skin flushing and, rarely, hepatotoxicity when used in pharmacological doses (1 g/day to 5 g/day). Hepatotoxicity manifests as a spectrum that includes mild elevation of liver enzymes, hepatic steatosis, hepatic necrosis, and, rarely, hepatic failure. Treatment includes discontinuation of niacin, and usually the liver enzymes return to normal. The smallest dose of niacin that leads to hepatotoxicity described in the literature is 1 g/day [2]. Our patient ingested around 300 mg of niacin daily (30 mg/can × 10 cans/day), making this the smallest reported dose that induced niacin toxicity.

Vitamin B_12 _is involved in nucleic acid metabolism, the formation of red blood cells (RBCs), and myelin synthesis and repair. It has a very low potential for toxicity even when taken in large doses. This does not imply that it has no adverse effects. Because data on the toxic profile of vitamin B_12 _are limited, consumers, especially pregnant women, should be cautious when ingesting large amounts of vitamin B_12_.

Folic acid is required for DNA synthesis, RBC synthesis, and cell growth. Since it is water-soluble and regularly excreted by the body, toxicity as a result of excessive ingestion does not commonly occur. According to the National Academy of Sciences, the daily intake of folic acid in adults should not exceed 1000 μg. Very high doses (> 15,000 μ g/day) can cause stomach problems, sleep problems, skin reactions, and seizures.

Citicoline functions as a neuro-protective agent. It exhibits a very low toxicity profile in humans. In a short-term, placebo-controlled study that compared citicoline to placebo, transient headaches occurred in patients in the citicoline group compared to the placebo group. No changes or abnormalities were observed in hematologic, biochemistry, liver function, or neurological tests in patients in that study [3].

Taurine is a normal metabolite in humans that is involved in the modulation of neuronal excitability, membrane stabilization, production of bile salts, and the detoxification of certain xenobiotics. There have been no adequate studies done to observe toxicity and/or carcinogenicity linked to taurine ingestion.

Caffeine functions as a psychoactive stimulant and a mild diuretic in humans. In large amounts, and especially over extended periods of time, caffeine can lead to a condition known as caffeinism [4,5]. Caffeinism refers to a wide range of symptoms that include anxiety, insomnia, headaches, respiratory alkalosis, and palpitations. Increased use over time can lead to peptic ulcers and gastroesophageal reflux disease. Acute caffeine intoxication (> 300 mg/day) leads to serious symptoms that include restlessness, arrhythmias, and psychomotor agitation. Extremely large doses of caffeine can cause acute psychosis, rhabdomyolysis, and even death. The treatment of severe caffeine intoxication is generally supportive, and dialysis may be required if the levels of caffeine are extremely high.

Tyrosine is involved in the synthesis of neurotransmitters in the brain. It has the potential for very low toxicity, and there have been very few reports of toxicity reported, none of which involved hepatotoxicity.

Phenylalanine is converted to tyrosine in the body and serves the same function as tyrosine. Toxicity symptoms include increased blood pressure, emotional agitation, insomnia, and headaches.

Malic acid is important in boosting immunity, maintaining oral health, and reducing the risk of poisoning from a build-up of toxic metals. There are no known reported contraindications or toxicities linked to malic acid.

D-Glucurono-γ-lactone is a natural metabolite of glucose and regulates the formation of glycogen. There is very little known about the toxicity of this substance.

## Conclusion

After having fairly ruled out other etiologies for our patient's spontaneously reversible severe hepatitis, and after carefully reviewing the toxicity profiles of the ingredients present in the energy drink she consumed, we postulated that in the face of excessive and prolonged consumption of this energy drink, the main ingredient most likely responsible for the development of acute hepatitis in this patient is vitamin B_3 _(niacin). Although her total ingestion of niacin was 300 mg/day, which is well below the quantity expected to cause toxicity, there is very little known about the toxicity profiles of some of the other compounds or about the interactions between them, which should be further studied. Additionally, since these energy drinks are not regulated by the U.S. Food and Drug Administration, the precise quantities of the ingredients and the interactions between them are not known. The daily increased consumption of this drink over an extended period of two weeks would also have contributed to the adverse effects she experienced. In conclusion, energy drinks are not traditional food products and contain some constituents that, while not unique to these products, are present in much higher concentrations than are found in other food products and/or natural foods. Hence the excessive ingestion of these drinks should be avoided.

## Abbreviations

ALT: alanine aminotransferase; AST: aspartate aminotransferase; CT: computed tomography; ED: Emergency Department; INR: international normalized ratio.

## Consent

Written informed consent was obtained from the patient for publication of this case report and any accompanying images. A copy of the written consent is available for review by the Editor-in-Chief of this journal.

## Competing interests

The authors report no financial relationships or conflicts of interest regarding the content herein.

## Authors' contributions

AV, SN, and AW analyzed and interpreted the patient data regarding the patient's presentation. AV was instrumental in obtaining informed consent from the patient and also in the preparation of the manuscript. All authors read and approved the final manuscript.
